# Editorial: Rising stars in drugs outcomes research and policies: 2021

**DOI:** 10.3389/fphar.2022.1076513

**Published:** 2022-11-25

**Authors:** Robert L. Lins, Fariba Ahmadizar, Tanveer Khan

**Affiliations:** ^1^ University of Antwerpen, Antwerpen, Belgium; ^2^ Department of Data Science and Biostatiscs, Julius Global Health, University Medical Center Utrecht, Utrect, Netherlands; ^3^ Drugs Control and Traditional Medicines Division, National Institute of Health, Islamabad, Pakistan

**Keywords:** rising stars, drugs, outcome research, comparison of treatments, meta-analysis, cost-effectiveness

A major part of scientific research is carried out by graduate students, postdoctoral researchers, and faculty who do not yet have permanent employment with an institute or company (Bankston et al., 2020). It is important to encourage early career reseachers as the scientific community needs new talents, skills and ideas to continue to flourish (Heggeness et al., 2017). Consequently, they should be supported to become an important part of the current and future scientific community. This group is also considerably more diverse in terms of gender and ethnicity than the rest of the research enterprise (Nikaj et al., 2018). Several universities and other groups have taken initiatives to help these young researchers with the challenges they encounter. Recommendations were proposed for empowerment of early career researchers (Kent et al., 2022) and more support for research in Low and Middle Income Countries (LMICs) is provided by groups like HTAi (Innovation through Health Technology Assessment) and MURIA (Medicine Utilisation in Africa) for greater research into medicine use (Massel et al., 2017).

Frontiers’ Drugs Outcomes, Research, and Policies explores the evaluation of drugs, advanced therapies and medical devices containing drugs in real life conditions, following their approval by regulatory authorities and access to the market.

Comparisons between drugs are increasingly used to assist decision makers in getting the biggest value for money within cost-constrained healthcare budgets. Drug cost are sometimes a small proportion of overall cost of illness but can account for up to 60–80% of total healthcare expenditure in LMICs—much of which is out of pocket. In addition, in high income countries most new medicines are targeted at cancers/orphan diseases at high prices. In these situations, the costs of medicines are a considerable proportion of the total costs of these diseases and are driving increasing medicine expenditure world wide. So these costs need to be carefully managed especially in countries with Universal Health Care. This is where good new young researchers can play a key role going forward.

We are delighted to present the Frontiers in Pharmacology “Rising Stars in Drugs Outcomes Research and Policies: 2021” series of article collections. Recognizing the future leaders in Drugs Outcomes Research is fundamental to safeguarding tomorrow’s driving force in innovation. This Research Topic is part of the Rising Stars in Pharmacology series.

The aim of this collection is to showcase the high-quality work of early career researchers within 10 years of PhD or MD completion across the entire breadth of Drugs Outcomes Research and Policies, and present advances in theory, experiment, and methodology with applications to compelling problems.

In total, 13 manuscripts were submitted, of which 8 were accepted for publication after the peer review process, seven from early career authors coming from China and one from Thailand.

Two articles studied cost-effectiveness of treatments for osteoarthritis and breast cancer. Three meta-analyses, of which one network meta-analysis compared efficacy and safety of treatments. The last two studies investigated the impact of disease on screening utilization and of a procurement policy on access to treatment.

Luksameesate et al. evaluated the cost-effectiveness of additional knee osteoarthritis treatments compared to standard treatment reimbursed by the major health insurance payer in Thailand. They showed that crystalline glucosamine sulfate and etoricoxib added to standard knee osteoarthritis treatment were cost-effective at the willingness-to-pay threshold in Thailand. Additionally, early initiation of crystalline glucosamine sulfate would be less costly and more effective than delayed treatment or standard treatment alone.

In another cost-effectiveness study, Shu et al. investigated the incremental cost-effectiveness ratio (ICER) of olaparib as maintenance therapy in patients with platinum-sensitive relapsed ovarian cancer and a BRCA1/2 mutation in China. Also using a Markov model, the findings from the present analysis suggest that olaparib might be cost-effective in these patients. This echos findings in real-life in Sweden where the outcomes seen in practice mirror those seen in the clinical trials - which is often not the case espeically if real-life patients are older/more co-morbid than those in RCTs (Eriksson et al., 2018).

Zhang et al. performed a network meta-analysis of the efficacy and safety of anti-vascular endothelial growth factor (VEGF) monotherapies for neovascular age-related macular degeneration. They found that the visual efficacy of four individual anti-VEGF drugs is comparable, but several statistically significant differences were observed. Considering special regimens. The authors conclude that, based on the premise of equivalent efficacy and safety, the optimal choice of anti-VEGF monotherapies seems mandatory to obtain maximal benefit.

Due to an increase in drug resistance, the eradication rate of Helicobacter Pylori with empirical therapy has declined. A systematic review and meta-Analysis, by Ma et al. determined whether tailored therapy is superior to empirical therapy. Tailored therapies might lead to better eradication rates than empirical therapies in first-line therapy. There might be no obvious advantage in second-line or third-line treatment. Due to the high heterogeneity, the results should be interpreted with caution.

In a meta-analysis, investigating the efficacy and safety of thalidomide, a fetal hemoglobin inducer, for treating patients with transfusion-dependent β-thalassemia (TFT), Lu et al. showed remarkable efficacy of thalidomide with an overall response rate (ORR) of 83% and pooled complete response rate (CRR) of 52%. The response to thalidomide did not show any statistically significant relationship with genetic mutations. They conclude that thalidomide is a relatively safe and effective therapy to reduce blood transfusion requirements and increase Hb level in patients with β-thalassemia.

Lv et al. found that in older adults with Alzheimer’s disease and related dementias (ADRD) in the Medicare population, ADRD was significantly associated with colorectal cancer screening utilization and knowledge. This study also identified health disparities in race/ethnicity, gender, and urban/rural residence.

Yuan et al. tackled the problem of cost-related access barriers to antiviral medications for hepatitis B virus (HBV). They found that national volume-based procurement (NVBP) piloted in China is effective for reducing price and total expenditures and improving drug utilization, which is especially important for HBV patients who need constant access to antiviral therapies.

In an online survey evaluating physicians’ ability to detect prescription of potentially inappropriate medication (PIM) to older patients, by Yuan et al. approximately 40% of PIM were recognized, suggesting an insufficient level of knowledge about appropriate prescribing.

Drugs Outcomes Research is a growing discipline in the health care area, and collaborative research with academic institutions and key opinion leaders is extremely essential to provide arguments for effective health care resource allocation (Duttagupt, 2010).

We conclude that in this Research Topic, a variety of articles has been presented, that provide great insight in drug efficacy, safety, health economics, health policies with promising outcomes and future perspectives. As Editors, we would like to thank all the contributing authors and the editorial office in Frontiers in Pharmacology for their excellent editing support.
